# N-terminal truncation of PhaC_BP-M-CPF4_ and its effect on PHA production

**DOI:** 10.1186/s12934-024-02329-w

**Published:** 2024-02-15

**Authors:** Soon Zher Neoh, Hua Tiang Tan, Chanaporn Trakunjae, Min Fey Chek, Pilanee Vaithanomsat, Toshio Hakoshima, Kumar Sudesh

**Affiliations:** 1 Ecobiomaterial Research Laboratory, School of Biological Sciences, Universiti Sains Malaysia, 11800 USM Pulau Pinang, Penang Malaysia; 2 Structural Biology Laboratory, Nara Institute of Science and Technology, 8916-5 Takayama, Ikoma, Nara 630-0192 Japan; 3 Kasetsart Agricultural and Agro-Industrial Product Improvement Institute (KAPI), Kasetsart University, Bangkok, 10900 Thailand

**Keywords:** Polyhydroxyalkanoate synthase, Poly(3-hydroxybutyrate-*co*-3-hydroxyhexanoate), N-terminal truncation, Molecular weights, *phaC* gene expression, PHA granule morphology

## Abstract

**Background:**

Among the polyhydroxyalkanoate (PHA), poly[(*R*)*-*3-hydroxybutyrate*-co-*(*R*)*-*3-hydroxyhexanoate] [P(3HB*-co-*3HHx)] is reported to closely resemble polypropylene and low-density polyethylene. Studies have shown that PHA synthase (PhaC) from mangrove soil (PhaC_BP-M-CPF4_) is an efficient PhaC for P(3HB*-co-*3HHx) production and N-termini of PhaCs influence its substrate specificity, dimerization, granule morphology, and molecular weights of PHA produced. This study aims to further improve PhaC_BP-M-CPF4_ through N-terminal truncation.

**Results:**

The N-terminal truncated mutants of PhaC_BP-M-CPF4_ were constructed based on the information of the predicted secondary and tertiary structures using PSIPRED server and AlphaFold2 program, respectively. The N-terminal truncated PhaC_BP-M-CPF4_ mutants were evaluated in *C. necator* mutant PHB^−^4 based on the cell dry weight, PHA content, 3HHx molar composition, molecular weights, and granule morphology of the PHA granules. The results showed that most transformants harbouring the N-terminal truncated PhaC_BP-M-CPF4_ showed a reduction in PHA content and cell dry weight except for PhaC_BP-M-CPF4_ G8. PhaC_BP-M-CPF4_ G8 and A27 showed an improved weight-average molecular weight (*M*_w_) of PHA produced due to lower expression of the truncated PhaC_BP-M-CPF4_. Transformants harbouring PhaC_BP-M-CPF4_ G8, A27, and T74 showed a reduction in the number of granules. PhaC_BP-M-CPF4_ G8 produced higher *M*_w_ PHA in mostly single larger PHA granules with comparable production as the full-length PhaC_BP-M-CPF4_.

**Conclusion:**

This research showed that N-terminal truncation had effects on PHA accumulation, substrate specificity, *M*_w_, and granule morphology. This study also showed that N-terminal truncation of the amino acids that did not adopt any secondary structure can be an alternative to improve PhaCs for the production of PHA with higher *M*_w_ in mostly single larger granules.

**Supplementary Information:**

The online version contains supplementary material available at 10.1186/s12934-024-02329-w.

## Background

Polyhydroxyalkanoate (PHA) is one of the bioplastics that are capable of replacing the current commercial petroleum-based plastic in certain applications with the advantage of being biodegradable at the same time [1, 2]. PHAs usually accumulate in the cytoplasm of most bacteria and archaea under the condition where there is an excess amount of carbon and limited nutrients like nitrogen [3]. Among the PHAs, poly(3-hydroxybutyrate-*co*-3-hydroxyhexanoate) [P(3HB-*co*-3HHx)] is known to have properties similar to low-density polyethylene (LDPE) and polypropylene (PP) [2]. The properties of the P(3HB-*co*-3HHx) produced vary depending on the molar composition of the 3HHx [4].

Among the genes involved in PHA biosynthesis, *phaC* is the most important gene because it encodes for PHA synthase (PhaC), the key enzyme in PHA biosynthesis by polymerizing the monomeric substrates [5]. PhaC determines the properties of the PHA produced as PhaCs can have different substrate specificities toward different monomers. The incorporation of different monomer compositions will affect the properties of the PHA produced [5–10]. Additionally, PhaCs play a role in determining the molecular weights of the PHA produced, as different PhaCs accumulate PHAs of different molecular weights. Molecular weights can affect the mechanical properties of the PHA produced [11–16]. Generally, it was reviewed that the higher the 3HHx molar composition, the lower the molecular weights of the P(3HB-*co*-3HHx) produced [4]. However, it was reported that although PhaC from *Chromobacterium* sp. USM2 (PhaC_*C*s_) had lower 3HHx incorporation compared to PhaC isolated from mangrove soil metagenome (PhaC_BP-M-CPF4_), the molecular weights of the P(3HB-*co*-3HHx) produced by PhaC_*C*s_ was very much lower compared to that of PhaC_BP-M-CPF4_ [16]. This report further highlights the importance of PhaCs in determining the molecular weights of the PHA produced regardless of its monomer composition.

The criterion of an efficient PhaC for the production of P(3HB-*co*-3HHx) is the ability to incorporate the desired amount of 3HHx molar composition while not compromising the weight-average molecular weight (*M*_w_). Higher *M*_w_ PHA with certain amount of 3HHx is desired as it contributes to better physical and mechanical properties like better tensile strength and extension to break [17, 18]. Having the right 3HHx molar composition is also necessary as it can lower the melting temperature of the PHA, lowering the processing temperature. However, there is just a handful of efficient PhaCs for the production of P(3HB-*co*-3HHx) like PhaC_BP-M-CPF4_, PhaC from *Rhodococcus aetherivorans* I24 (PhaC_*Ra*_), PhaC from *Aeromonas hydrophila* 4AK4 (PhaC_*Ah*_), PhaC from *Aeromonas* sp. LFM897 (PhaC_*A*s_), and PhaC from *Aeromonas caviae* (PhaC_*Ac*_) have shown promise in this regards [8, 19–22]. Besides isolating a naturally-evolved PhaC with broad substrate preferences, protein engineering including single and multiple point mutations were employed on PhaC_*Ac*_ for better 3HHx incorporation and production of higher molecular weight copolymers of P(3HB-*co*-3HHx) [23, 24]. Apart from being able to accumulate P(3HB-*co*-3HHx) with desired 3HHx molar composition and high molecular weights, accumulating PHAs in single large granules is also desired as it will ease the downstream processes of PHA extraction and purification [25, 26]. Currently, metabolic engineering of PhaPs is the most common way of manipulating the number and size of PHA granules. Previous studies had shown that overexpression of *phaP*s can lead to the increment in number of smaller PHA granules, and expression of *phaP*s from various organisms in different hosts may lead to larger PHA granule and affecting the *M*_w_ of PHA produced [9, 27].

The structure of a PhaC can be divided into 2 parts: the N-terminal and the C-terminal domains. Early researches suggested that based on the multiple sequence alignment of well-reported PhaCs, the N-terminal region of a PhaC is approximately the first 100 amino acids as they are highly variable and flexible [5, 6]. Furthermore, the N-terminal region is also highly flexible as it hinders the crystallization process of protein crystals [6]. However, the crystal structure of PhaCs from *Cupriavidus necator* H16 (PhaC_*Cn*_) and PhaC_*C*s_ later suggested that the N-terminal of PhaCs may vary between different PhaCs. The N-terminal of PhaC_*Cn*_ and PhaC_*C*s_ were suggested to be the first 200 and 174 amino acids respectively, as the PhaCs were able to crystalize and remain stable without them [28]. On the other hand, the C-terminal of PhaCs was reported to consist of the cap subdomain, substrate entrance channel, and product egress channel [6, 29–32]

To date, there are reports regarding the N-terminal of a PhaC affecting the substrate specificity and molecular weights of the PHA produced [33, 34]. Studies on chimeric PhaC of PhaC_*Cn*_ with various N-terminal lengths of PhaC from *Pseudomonas aeruginosa* (PhaC_*Pa*_) were performed. As a result, chimeric PhaC of PhaC_*Cn*_ and PhaC_*Pa*_ was observed to incorporate 3HHx whereas the wild-type PhaC_*Cn*_ do not [35]. In addition, Lim and co-workers reported that the N-terminal of PhaC was needed for the dimer formation of PhaCs [36]. Despite decades of advancements in PhaC research, the exact functional roles of N-terminal domain of PhaC remain elusive. In this study, the N-terminal of PhaC_BP-M-CPF4_ will be truncated according to its predicted secondary and tertiary structure and evaluated based on its incorporation of 3HHx monomer, the molecular weights of the PHA produced, and granule morphology for a better PhaC in the production of P(3HB-*co*-3HHx) copolymer. Gene expression study was performed to study how N-terminal region truncation affects the molecular weights of P(3HB-*co*-3HHx) produced.

## Methods

### Bacterial strain, plasmid, and PHA biosynthesis condition

In this study, the PHA-negative mutant strain of *C. necator*, PHB^−^4 was used as the host strain for the evaluation of the N-terminal truncated PhaC_BP-M-CPF4_. The N-terminal truncated *phaC*_BP-M-CPF4_ mutants (G8; 8–551, A27; 27–551, T74; 74–551, and D104; 104–551) were inserted into previously modified pBBR1MCS-2 plasmid which harbours the *phaC1* promoter from *C. necator* (Table 1) [8]. The *C. necator* transformants were first pre-cultured in nutrient-rich (NR) media until OD_600nm_ reaches 4–4.5 before transferring 3% (v/v) of the preculture into 50 mL mineral medium (MM) [11, 20]. The carbon sources used in this study were 1% (v/v) of crude palm kernel oil (CPKO) or 2% (w/v) of fructose supplemented by 0.054% (w/v) of urea as the nitrogen source. Kanamycin was added during the cultivation of *C. necator* transformants to the final concentration of 50 µg/mL. The cultures were then shaken at 200 rpm, 30 °C for 48 h. After 48 h, the cultures were harvested. The harvested cells were then lyophilized before use for further analysis [37].

**Table 1:** List of strains, and plasmids used in this study

Strain or plasmid	Description	Reference or source
Strains		
*C. necator* PHB^−^4	*C. necator* with nonsense mutation of *phaC1*_*Cn*_ at the 107th amino acid	DSM 541
*E. coli* DH5α	General cloning strain. F^−^ l^−^ deoR supE44 hsdR17(rK^−^ mK^+^) phoA *rec*A1 *end*A1 *gyr*A96 *thi*-1 relA1 D(*lac*ZYA-*arg*F) U169 f80d*lac*ZDM15	Toyobo
*E. coli* S17-1	*E. coli* strain for transconjugation of the plasmid into *C. necator. rec*A, *tra* genes of plasmid RP4 integrated into the chromosome, auxotrophic for proline and thiamin	[38]
Plasmids		
pBBR1MCS2	Broad-host-range cloning vector Km^r^, *mob, lacZ*α	[39]
pBBR1-Pro_*Cn*_	pBBR1MCS2 plasmid containing *phaC1* promoter from *C. necator*	[8]
pBBR1-*C*_BP-M-CPF4_	pBBR1MCS2 plasmid harbouring the *phaC1* promoter of *C. necator* controlling the expression of *phaC*_BP-M-CPF4_	[8]
pBBR1-*C*_BP-M-CPF4_ G8	pBBR1MCS2 plasmid harbouring the *phaC1* promoter of *C. necator* controlling the expression of *phaC*_BP-M-CPF4_ 8–551 amino acids	This study
pBBR1-*C*_BP-M-CPF4_ A27	pBBR1MCS2 plasmid harbouring the *phaC1* promoter of *C. necator* controlling the expression of *phaC*_BP-M-CPF4_ 27–551 amino acids	This study
pBBR1-*C*_BP-M-CPF4_ T74	pBBR1MCS2 plasmid harbouring the *phaC1* promoter of *C. necator* controlling the expression of *phaC*_BP-M-CPF4_ 74–551 amino acids	This study
pBBR1-*C*_BP-M-CPF4_ D104	pBBR1MCS2 plasmid harbouring the *phaC1* promoter of *C. necator* controlling the expression of *phaC*_BP-M-CPF4_ 104–551 amino acids	This study

### PHA synthase structure prediction

The amino acid sequence of PhaC_BP-M-CPF4_ (accession no. AXB72506.1) was first obtained from National Center for Biotechnology Information (NCBI) (https://www.ncbi.nlm.nih.gov/protein/1423452970). The tertiary structure and secondary structure of amino acid sequence obtained was then predicted using AlphaFold2 program (https://www.ebi.ac.uk/Tools/sss/fasta/) and PSIPRED server (http://bioinf.cs.ucl.ac.uk/psipred/), respectively.

### Construction of *C. necator* PHB^−^4 mutant harbouring N-terminal truncated PhaC_BP-M-CPF4_

The full nucleotide sequence of PhaC_BP-M-CPF4_ (accession no. MF431721.1) from a plasmid in a previous study (pBBR1-*C*_BP-M-CPF4_) was used as the template for polymerase chain reaction (PCR) to amplify the N-terminal truncated mutants (G8, A27, T74, and D104) using Q5® High-Fidelity 2 × Master Mix (New England Biolabs Inc., USA). The primers designed for the amplification of the N-terminal truncated PhaC_BP-M-CPF4_ mutants were listed in Table 2. For all the N-terminal truncated mutants, sequence of “ATG” was added in the starting of the sequence as the start codon which codes for methionine. The PCR products of the N-terminal truncated PhaC_BP-M-CPF4_ mutants were then digested using FastDigest *Hin*dIII and *Apa*I restriction enzymes (Thermo Scientific, USA). The vector of this study, pBBR1-Pro_*Cn*_ was also digested with similar restriction enzymes. The expression of *phaC*_BP-M-CPF4_ with its N-terminal region truncated mutants were controlled by the native *phaC1* promoter of *C. necator* [8]. The PCR products were ligated with the digested pBBR1-Pro_*Cn*_ using DNA Ligation Kit Ver.2.1 (Takara Bio Inc., Japan) according to the manufacturer’s protocol, and the resultant plasmids were transformed into *C. necator* PHB^−^4 via transconjugation with *Escherichia coli* S17-1 [40].

**Table 2:** List of primers used in this study

Primer	Sequence	Source or reference
*Hin*dIII_RBS_PhaC_BP-M-CPF4_G8_Fwd	AGT **AAGCTT** CAAAGGAGGGAAAGT ATGGGGAAAACAGGTGATTTGTGGTCAT	This study
*Hin*dIII_RBS_PhaC_BP-M-CPF4_A27_Fwd	AGT **AAGCTT** CAAAGGAGGGAAAGT ATGGCCGCGGCACAGATTCAGCA	This study
*Hin*dIII_RBS_PhaC_BP-M-CPF4_T74_Fwd	AGT **AAGCTT** CAAAGGAGGGAAAGT ATGACGGAAAACATGGCCGCCGA	This study
*Hin*dIII_RBS_BP-M-CPF4_D104_Fwd	AGT **AAGCTT** CAAAGGAGGGAAAGT ATGGATCCCGATCTGCACGAAC	This study
*Apa*I_PhaC_BP-M-CPF4_Rev	ATT **GGGCCC** CTACTTCTCCAAAACGTACGT	This study
qPCR_ PhaC_BP-M-CPF4_Forw	CACCACCCACCAGGATTTCAGC	This study
qPCR_ PhaC_BP-M-CPF4_Rev	GTAATTCCATAGCAGGTCATTGGCCCG	This study
qPCR_16S_Forw	GTGGCGAACGGGTGAGTAATACATCG	This study
qPCR_16S_Rev	CCAGCTACTGATCGTCGCCTTGG	This study

The bold letters represent the restriction site. The ribosome binding site is underlined

### Analysis of the PHA produced by *C. necator* PHB^−^4

The PHA content and molar composition of the PHA produced by the *C. necator* PHB^−^4 transformants harbouring full-length PhaC_BP-M-CPF4_ and its N-terminal truncated mutants were determined using gas chromatography (GC) [41]. The GC was first calibrated using chloroform extracted homopolymer of P(3HB) and copolymer of P(3HB-*co*-11 mol% 3HHx). A total of 15 to 20 mg of lyophilized cells were weighed and subjected to methanolysis by heating them to 100 °C for 140 min in the methanolysis solution containing 15% (v/v) sulfuric acid and 85% (v/v) methanol. Caprylic acid methyl ester (CME) was used as an internal standard to calculate the PHA content in the lyophilized cells. The PHA produced were analyzed using SHIMADZU GC-2010 Plus (Shimadzu, Japan) equipped with AOC-20i Auto-injector (Shimadzu, Japan), Rtx-1 GC column (Restek, USA) and flame ionization detector with nitrogen gas as carrier gas. The injector and detector temperatures were set to 270 °C and 280 °C respectively. The column temperature was initially set at 70 °C but gradually increased to 280 °C at a rate of 10 °C/min.

The PHA accumulated in the lyophilized cells was extracted using the solvent extraction method. The lyophilized cells were weighed, and chloroform was added to a ratio of 1 g lyophilized cells: 100 mL chloroform. The mixture was stirred for 72 h at room temperature. The mixture was then filtered using filter paper to remove the cell debris and the chloroform-dissolved PHA was precipitated using chilled methanol. The weight-average molecular weight (*M*_w_), number-average molecular weight (*M*_n_), and polydispersity index (PDI) of the PHA accumulated were determined through gel permeation chromatography (GPC) analysis. About 1 mg of PHA was dissolved in 1 mL of HPLC-grade chloroform and injected into the Agilent Technology 1200 Series High-Performance Liquid Chromatography (HPLC) system (Agilent, USA) equipped with a refractive index detector. The columns were TSK guard column H_HR_-H and TSKgel GMH _HR_-H (Tosoh, Japan). The flow rate was set to 0.8 mL/min and the column temperature was maintained at 40 °C [42].

### Total RNA extraction and quantitative PCR (qPCR)

The effect of N-terminal truncation on the PhaC expression was also analyzed using qPCR analysis. The *C. necator* PHB^−^4 harbouring the full-length of PhaC_BP-M-CPF4_, and its N-terminal truncated mutants were cultured in PHA biosynthesis condition as described in previously using 2% (w/v) of fructose and 0.054% (w/v) of urea as the nitrogen source. Unlike previously, the bacterial cells were harvested at 24 h [43]. The bacterial cells were washed and resuspended in 10 mL of sterilized deionized water. About 1 mL of the suspension was centrifuged to obtain its pellet. The pellet was then resuspended in 200 µL of enzymatic lysis buffer containing 20 mg/mL of lysozyme dissolved in a mixture of 20 mM Tris–Cl (pH 8.0), 2 mM sodium EDTA, and 1.2% Triton® X-100. The suspension was incubated for 1 h at 37 °C. After that, 25 µL of Proteinase K (Thermo Scientific, USA) was added and mixed by pipetting. The mixture was incubated at 56 °C for 30 min. The total RNA was separated from other cellular components using TRI REAGENT®-RNA/DNA/PROTEIN ISOLATION REAGENT and done according to the manufacturer’s protocol and further purified using RNeasy® Mini Kit according to the modified manufacturer’s protocol. The total RNA concentration was determined using BioSpectrometer® (Eppendorf, Germany) equipped with Eppendorf µCuvette G1.0 (Eppendorf, Germany). The total RNA extracted was stored at − 80 °C before use.

A total of 1000 ng of the extracted total RNA was converted to cDNA using QuantiNova™ Reverse Transcription Kit (Qiagen, Germany) according to the manufacturer’s protocol. Primer efficiency and qPCR analysis were done with QuantiNova™ SYBR Green PCR Kit (Qiagen, Germany) using CFX96™ Real-Time System (Biorad, USA) according to the manufacturer’s protocol before determining the expression of *phaC*_BP-M-CPF4_, and its N-terminal truncated mutants. The primers used for qPCR analysis were listed in Table 2. The condition of the qPCR was optimized until primer efficiency values were in the range of 90 to 110%. A total of 75 ng of cDNA was mixed with 0.7 µL of 10 µM of forward and reverse primers, 5 µL of SYBR Green PCR Master Mix, and RNase-free water was added until the total volume of 10 µL. The PCR reaction was done at 95 °C for 5 min as the initial activation step, followed by 40 cycles of denaturation of the cDNA at 95 °C for 10 s, annealing, and extension at 60 °C for 30 s. The data obtained from the qPCR were analysed using the relative quantification method where it was expressed as fold change between the *phaC*_BP-M-CPF4_ (control) and its N-terminal truncated mutants. 16S rRNA was used as an internal control. The cycle threshold (Ct) was obtained, and the relative comparison of the truncated mutants to the full-length *phaC*_BP-M-CPF4_ was analysed using the 2^−ΔΔCt^ formula [44, 45].

### mRNA secondary structure prediction

The mRNA secondary structure of the transcribed *phaC*_BP-M-CPF4_ was also determined. First, the transcription start site in the *C. necator* promoter from the pBBR1-Pro_*Cn*_ was predicted using SAPPHIRE online software (https://sapphire.biw.kuleuven.be/index.php) [46]. The nucleotide sequence starting from transcription start site until the end of full-length *phaC*_BP-M-CPF4_ was converted to mRNA sequence using DNA to mRNA to Protein Converter (https://skaminsky115.github.io/nac/DNA-mRNA-Protein_Converter.html). The mRNA sequence obtained was input into RNAfold online software (http://rna.tbi.univie.ac.at/cgi-bin/RNAWebSuite/RNAfold.cgi) for predicting its secondary structure [47].

### Determination of the number and diameter of granules in each bacterial cell

The change in the granule morphology of the *C. necator* PHB^−^4 transformants harbouring the full-length of PhaC_BP-M-CPF4_ and its N-terminal truncated mutants was also determined using transmission electron microscopy (TEM). The *C. necator* PHB^−^4 harbouring the full-length of PhaC_BP-M-CPF4_, G8, A27, and T74 were cultured in PHA biosynthesis condition as described previously using 2% (w/v) of fructose supplemented by 0.054% (w/v) of urea as nitrogen source and were harvested as described previously. The harvested bacterial cells were fixed with McDowell-Trump fixatives at 4 °C for at least 24 h [48]. The fixed bacterial cells were treated with 1% osmium tetroxide (OsO_4_) for 1 h at room temperature, followed by dehydration gradually using ethanol concentrations starting from 50%, 75%, 95%, and 100%, respectively, and finally into 100% acetone. The dehydrated bacterial cells were embedded in low-viscosity Spurr’s resin and cured at 60 °C for at least 48 h [49]. Ultrathin sections were prepared using ultramicrotome and mounted on a copper grid followed by staining using uranyl acetate and lead citrate. The samples were then viewed under Philip CM 12/STEM and JLM-2000FX11. The number of granules in each of the mutants were counted and analyzed. A pie chart showing the percentage of bacteria cells with their respective number of PHA granules per bacterial cell was plotted and the diameter of the respective PHA granules were determined based on the TEM images obtained for *C. necator* PHB^−^4 harbouring the full-length of PhaC_BP-M-CPF4_, G8, A27, and T74.

### Phase contrast microscopy analysis

Phase contrast microscopy was used to determine the live condition of the cultured *C. necator* PHB^−^4 transformants harbouring the full-length of PhaC_BP-M-CPF4_ and its N-terminal truncated mutants. The transformants were cultured in PHA biosynthesis condition as described previously. About 1 mL of the bacterial culture was collected. A drop of the culture was dropped and spread gently onto a clean glass slide followed by being gently covered with a clean cover slip. The bacteria were observed using an Olympus System Microscope Model BX41 phase contrast light microscope (Olympus, Japan). Observation of the bacterial sample was done using the objective lens with the lowest magnification (10×) to the highest magnification (100×). The PHA accumulated in the bacterial cells will be seen as transparent white granules under phase contrast microscopy.

### Statistical analysis

All the data obtained were noted as mean ± standard deviation. All the statistical analyses were performed using SPSS software (IBM SPSS Statistics 24). The statistical data obtained were compared using one-way analysis of variance (ANOVA) with Tukey post hoc comparison with p values < 0.05 considered as statistically significant.

## Results

### Generation of ***C. necator*** PHB^−^4 harbouring the N-terminal truncated PhaC_BP-M-CPF4_

**Fig. 1:**
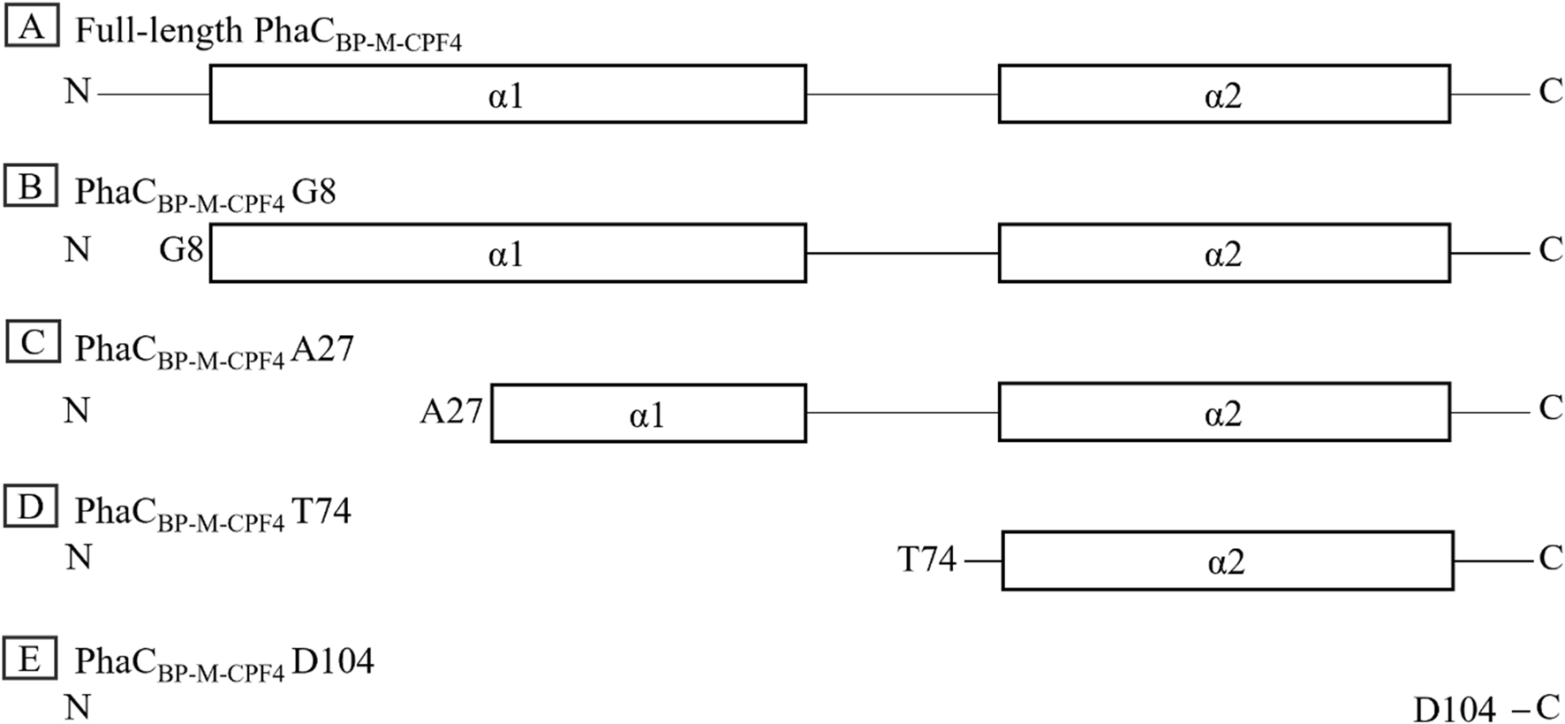
Illustration diagram of full-length PhaC_BP-M-CPF4_ and its N-terminal truncated mutants

To generate N-terminal truncated PhaC_BP-M-CPF4_, predicted tertiary and secondary structures of PhaC_BP-M-CPF4_ were employed and referred to design the truncation points. The tertiary N-terminal structure of PhaC_BP-M-CPF4_ was predicted using Alphafold2 program (Additional file 1: Fig. S1A). Due to the low pLDDT score on the first 13 amino acid residues of the predicted tertiary structure from Alphafold2 program, secondary structure from PSIPRED server was referred for the truncation point (Additional file 1: Fig. S1B). Based on the two predicted structures, a predicted secondary N-terminal structure of PhaC_BP-M-CPF4_ with combination of AlphaFold2 program and PSIPRED server was done (Additional file 1: Fig. S1C). Figure 1 illustrates the simplified diagram of the full-length PhaC_BP-M-CPF4_ and its N-terminal truncated mutants. As a result, the first seven amino acids were truncated since they did not adapt to any secondary helix structure, and it was designated as PhaC_BP-M-CPF4_ G8 (comprised of residues 8–551). In the second mutant, PhaC_BP-M-CPF4_ A27 (comprised of residues 27–551), the first 26 amino acid residues were truncated as it was extended from the adjacent pairing α2 helix based on the Alphafold2 structure (Additional file 1: Fig. S1A). In addition, secondary structure prediction from PSIPRED server (Additional file 1: Fig. S1B) indicated the α1-helix was separated into 2 distinct α-helices with a small gap at A27. Hence, A27 could be a good position for truncation to avoid structural disruption of the α1-helix. The third truncated mutant, PhaC_BP-M-CPF4_ T74 (comprised of residues 74–551), the N-terminal truncated mutant started from α2-helix where the first 73 amino acid residues comprised of α1-helix (Additional file 1: Fig. S1C). In the fourth N-terminal truncated mutant PhaC_BP-M-CPF4_ D104 (comprised of residues 104–551), the first 103 amino acids residues were truncated, which consist of α1, and α2-helices were truncated. In summary, this study evaluates the performances of the N-terminal truncated PhaC_BP-M-CPF4_ mutants on PHA production, PHA polymer’s properties, and PHA granule morphology.

### Production and molecular weights of P(3HB) and P(3HB-*co*-3HHx) by full-length PhaC_BP-M-CPF4_ and its N-terminal truncated mutants

Table 3 showed the PHA production of the full-length PhaC_BP-M-CPF4_ and its N-terminal truncated mutant PhaCs using *C. necator* PHB^−^4 as bacterial host. A total of 20 g/L of fructose or 10 g/L of crude palm kernel oil (CPKO) were used as the carbon source to produce P(3HB) and P(3HB-*co*-3HHx), respectively. There was no significant difference in the cell dry weight (CDW) of *C. necator* PHB^−^4 transformants expressing full-length PhaC_BP-M-CPF4_ and G8 when fructose was used as carbon source, which were 8.3 and 8.4 g/L respectively. A similar trend was observed when CPKO was used as the carbon source which was 11.3 and 10.2 g/L respectively. There were also no significant differences in the PHA content accumulated by *C. necator* PHB^−^4 harbouring full-length PhaC_BP-M-CPF4_ and G8 regardless of the carbon sources used. However, as more N-terminal regions were truncated in PhaC_BP-M-CPF4_ A27, T74 and D104, there was a significant reduction from 4.6 to 1.8 g/L and 56.5 to 0.5 wt% in the CDW and PHA content of the *C. necator* PHB^−^4 transformants when fructose was used as carbon source. Similarly, when CPKO was used as carbon source, there was a decrease from 7.5 to 1.8 g/L and 59.7 to 10.3 wt% in CDW and PHA content from PhaC_BP-M-CPF4_ A27 to D104 respectively. As for the residual biomass, there were no significant differences for *C. necator* PHB^−^4 harbouring the full-length PhaC_BP-M-CPF4_, G8, A27, and D104 ranging between 1.8 and 2.0 g/L when fructose was used as the carbon source. When CPKO was used as the carbon source, there were no significant differences between *C. necator* PHB^−^4 harbouring the full-length PhaC_BP-M-CPF4_, G8, and A27, ranging between 2.5 and 3.0 g/L. The residual biomass accumulated by *C. necator* PHB^−^4 harbouring PhaC_BP-M-CPF4_ T74 and D104 ranged between 1.6 and 1.7 g/L when CPKO was used as carbon source. When the bacteria cells were fed with CPKO, there was a gradual decrease in the 3HHx molar composition of the P(3HB-*co*-3HHx) produced from 6 to 1 mol% as more N-terminal region of the PhaC was truncated.

**Table 3:** PHA production of the *C. necator* PHB^−^4 transformants harbouring the full-length *phaC*_BP-M-CPF4_ or truncated *phaC*_BP-M-CPF4_ using fructose or CPKO with its molecular weights

Carbon source	Strain	Cell dry weight (g/L)	PHA content (wt%)	Residual biomass (g/L)	PHA composition (mol%)		Molecular weights (× 10^5^ Da)		
					3HB	3HHx	*M*_n_	*M*_w_	PDI
Fructose	PHB^−^4/ pBBR1-*C*_BP-M-CPF4_	8.3 ± 0.1^v^	78.0 ± 2.9^v^	1.8 ± 0.2^vw^	100 ± 0^v^	0 ± 0^v^	9.8 ± 0.5^v^	22.8 ± 0.1^v^	2.3 ± 0.1^v^
	PHB^−^4/pBBR1-*C*_BP-M-CPF4_ G8	8.4 ± 0.2^v^	81.9 ± 4.1^v^	1.5 ± 0.3^v^	100 ± 0^v^	0 ± 0^v^	11.2 ± 0.5^w^	26.1 ± 0.5^w^	2.3 ± 0.1^v^
	PHB^−^4/pBBR1-*C*_BP-M-CPF4_ A27	4.6 ± 0.1^w^	56.5 ± 2.9^w^	2.0 ± 0.2^vw^	100 ± 0^v^	0 ± 0^v^	10.9 ± 0.8^v^	26.9 ± 0.3^w^	2.5 ± 0.5^v^
	PHB^−^4/pBBR1-*C*_BP-M-CPF4_ T74	2.4 ± 0.0^x^	17.8 ± 0.4^x^	2.0 ± 0.0^w^	100 ± 0^v^	0 ± 0^v^	6.7 ± 0.1^x^	18.2 ± 0.1^x^	2.7 ± 0.0^w^
	PHB^−^4/pBBR1-*C*_BP-M-CPF4_ D104	1.8 ± 0.0^y^	0.5 ± 0.1^y^	1.8 ± 0.0^v^	100 ± 0^v^	0 ± 0^v^	ND	ND	ND
CPKO	PHB^−^4/ pBBR1-*C*_BP-M-CPF4_	11.3 ± 0.9^v^	75.8 ± 2.4^v^	2.7 ± 0.5^v^	94 ± 0^v^	6 ± 0^v^	3.7 ± 0.3^v^	9.3 ± 0.4^v^	2.5 ± 0.1^v^
	PHB^−^4/pBBR1-*C*_BP-M-CPF4_ G8	10.2 ± 0.3^v^	75.9 ± 2.7^v^	2.5 ± 0.3^v^	95 ± 0^w^	5 ± 0^w^	4.6 ± 0.1^w^	11.2 ± 0.2^w^	2.4 ± 0.0^v^
	PHB^−^4/pBBR1-*C*_BP-M-CPF4_ A27	7.5 ± 0.4^x^	59.7 ± 5.1^w^	3.0 ± 0.4^v^	96 ± 0^x^	4 ± 0^x^	5.7 ± 0.2^x^	14.1 ± 0.1^x^	2.5 ± 0.1^v^
	PHB^−^4/pBBR1-*C*_BP-M-CPF4_ T74	3.8 ± 0.1^y^	54.1 ± 5.4^w^	1.7 ± 0.1^w^	97 ± 0^y^	3 ± 0^y^	5.3 ± 0.1^y^	10.5 ± 0.0^y^	2.0 ± 0.0^w^
	PHB^−^4/pBBR1-*C*_BP-M-CPF4_ D104	1.8 ± 0.3^z^	10.3 ± 2.7^x^	1.6 ± 0.2^w^	99 ± 0^z^	1 ± 0^z^	ND	ND	ND

The bacterial cells were cultivated in 50 mL of MM supplemented with 0.054% (w/v) of urea as nitrogen source, 50 µg/mL kanamycin, 2% (v/v) of fructose or 1% (v/v) of CPKO as carbon source. The bacterial cells were shaken at 200 rpm for 48 h at 30 °C. The values reported are in means ± standard deviations of triplicate. The superscripts v, w, x, y, and z represent the significant differences in the GC and GPC data using one-way ANOVA (p < 0.05). Abbreviations: 3HB, 3-hydroxybutyrate; 3HHx, 3-hydroxyhexanoate; ND, not determined

As for the molecular weights, there was an increase in the *M*_w_ of both P(3HB) and P(3HB-*co*-3HHx) produced by the truncated PhaC_BP-M-CPF4_ G8 and A27 compared to the full-length PhaC_BP-M-CPF4_ (Table 3). The *M*_w_ of the PHA produced by PhaC_BP-M-CPF4_ G8 and A27 were 26.1 and 26.9 × 10^5^ Da for P(3HB), and 11.2 and 14.1 × 10^5^ Da for P(3HB-*co*-3HHx), respectively. For P(3HB), the *M*_n_ of the homopolymer produced ranged from 6.7 to 11.2 × 10^5^ with the PDI ranged from 2.3 to 2.7. For P(3HB-*co*-3HHx), the *M*_n_ ranged from 3.7 to 5.7 × 10^5^ while the PDI ranged from 2.0 to 2.5. The *M*_n_, *M*_w_, and PDI for both P(3HB) and P(3HB-*co*-3HHx) produced by PhaC_BP-M-CPF4_ D104 was not determined as the PHA accumulated was too low to be extracted for the analysis.

### Gene expression of N-terminal truncated PhaC_BP-M-CPF4_

**Fig. 2:**
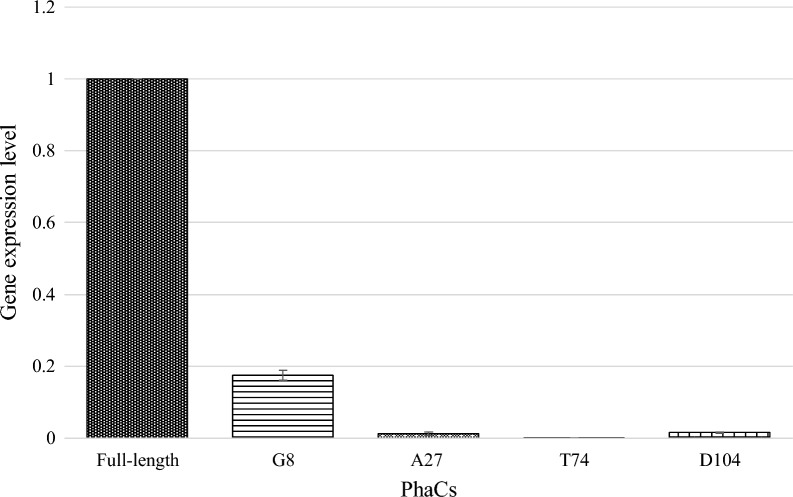
Gene expression of full-length PhaC_BP-M-CPF4_ compared to its N-terminal truncated mutants

Primer efficiency and melt curve analysis were performed to validate the primers designed for this study (Additional file 1: Fig. S2 and 3). Based on Fig. 2, there was a clear decrease in the gene expression level of the all the N-terminal truncated *phaC*_BP-M-CPF4_ mutants compared to its full-length. Compared to the full-length *phaC*_BP-M-CPF4_, there was a drop in gene expression level of more than 80% in all the N-terminal truncated PhaC_BP-M-CPF4_ mutants. Gene expression level of *phaC*_BP-M-CPF4_ G8 was the highest among all the N-terminal truncated *phaC*_BP-M-CPF4_ with about 0.17. The gene expression level of *phaC*_BP-M-CPF4_ A27 and D104 were low but comparable to each other ranging from 0.0124 to 0.0162 while *phaC*_BP-M-CPF4_ T74 was the lowest with just 0.0010. These results showed that truncation of the N-terminal region of *phaC*_BP-M-CPF4_ had effects on the gene expression or concentration of its corresponding mRNA. Based on the trends in Fig. 2, it can be suggested that the more N-terminal regions were truncated, the lower the expression of the N-terminal truncated *phaC*_BP-M-CPF4_ was. The decrease in the detectable mRNA concentration may also be due to its mRNA stability. In this study, the same promoter and RBS were used for the expression of full-length *phaC*_BP-M-CPF4_ and its N-terminal truncated mutants. This showed that the missing truncated N-terminal region might be necessary for better expression and/or mRNA stability of the *phaC*_BP-M-CPF4_ gene.

### Altered morphology of the PHA granules accumulated in the *C. necator* PHB^−^4 transformants

**Fig. 3:**
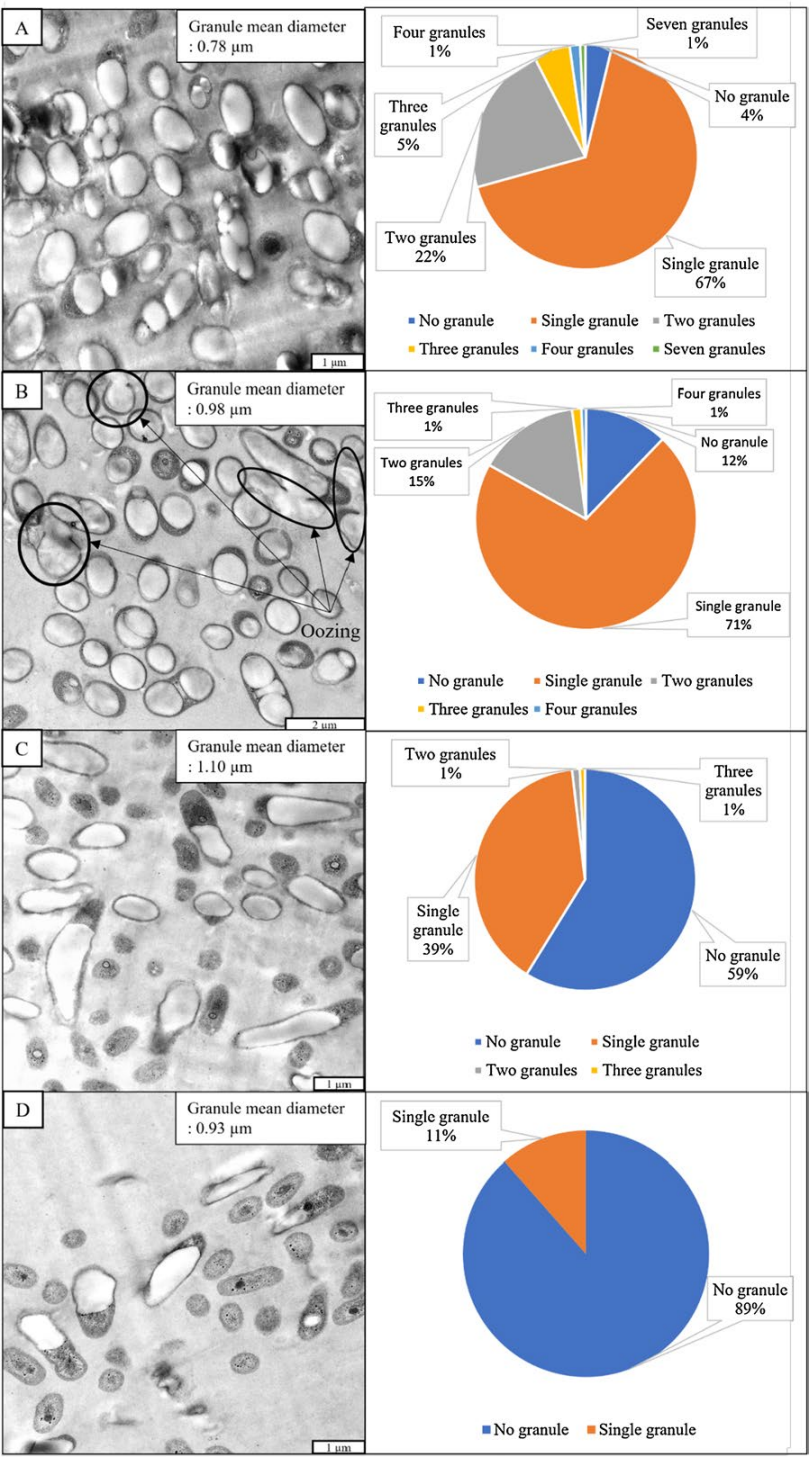
P(3HB) granule morphology of *C. necator* PHB^−^4 harbouring full-length of PhaC_BP-M-CPF4_ and its truncated mutants. TEM was used to view *C. necator* PHB^−^4 harbouring full-length of PhaC_BP-M-CPF4_, Magnification: 4000× (**A**), G8, Magnification: 3200× (**B**), A27, Magnification: 4000× (**C**), and T74, Magnification: 4000× (**D**). The pie charts representing the percentage of bacteria cell with their respective number of granules per bacterial cell were plotted. The total mean diameter of all the PHA granules were shown in the upper-right corner of TEM images. The oozing phenomenon is indicated using black circles. The TEM images shown represent the number of granules in each bacteria harbouring the full-length of PhaC_BP-M-CPF4_ and its truncated mutants

The P(3HB) granule morphology produced by the *C. necator* PHB^−^4 harbouring full-length PhaC_BP-M-CPF4_ and its N-terminal truncated mutants were examined using TEM. Figure 3 showed that the N-terminal of PhaC_BP-M-CPF4_ truncation resulted in a lesser number of PHA granules accumulated by the *C. necator* PHB^−^4 transformants. Based on pie charts shown in Fig. 3, there was an increase in the percentage of bacteria having single granules from full-length PhaC_BP-M-CPF4_ to G8. There was also a decrease in the percentage of bacterial cells having at least two PHA granules as more N-terminal regions of the PhaC_BP-M-CPF4_ were truncated from full-length PhaC_BP-M-CPF4_ to T74. This observation showed that there was clear evidence where the N-terminal truncation of PhaC_BP-M-CPF4_ affected the number of PHA granules accumulated in the bacterial mutants.

Besides having lesser PHA granules, based on Fig. 3, there were also an increased in the granule mean diameter of the PHA granules of from *C. necator* PHB^−^4 transformants harbouring full-length PhaC_BP-M-CPF4_ to A27 but decreased for *C. necator* PHB^−^4 transformants harbouring full-length PhaC_BP-M-CPF4_ T74. The granule mean diameter of the PHA granules increased from 0.78 (full-length PhaC_BP-M-CPF4_) to 1.10 µm (PhaC_BP-M-CPF4_ A27) but decreased to 0.93 µm (PhaC_BP-M-CPF4_ T74). This hinted that not only did the N-terminal region affect the number of granules formed but also the size of the PHA granules.

On the other hand, there was also an increase in the percentage of bacterial cells without any PHA accumulated as more N-terminal regions were truncated. Based on the pie chart in Fig. 3, the percentage of bacteria having no PHA granules increased from full-length PhaC_BP-M-CPF4_ to T74. In Fig. 3C, and D, the percentage of bacteria having no granule were even higher than the percentage of bacteria who had at least one PHA granules. The observation indicated that the N-terminal region of PhaC_BP-M-CPF4_ was also necessary for the production and accumulation of PHA in the bacteria. This speculation was demonstrated when 59% of *C. necator* PHB^−^4 transformants harbouring PhaC_BP-M-CPF4_ A27 showed no PHA accumulation, and the percentage further hiked to 89% in *C. necator* PHB^−^4 mutant harbouring PhaC_BP-M-CPF4_ T74.

Another interesting morphology observed from the TEM images was the fluidic nature of the PHA granules in the bacterial cells. In Fig. 3B, some of the PHA material was flowing out of or oozing from several granules (circled in black). The fluidic PHA material was not only seen flowing out of the granules but also appeared to be flowing/oozing out of the bacterial cells. Such behavior was not observed in any other samples examined in this study.

## Discussion

It is preferable to have a PhaC that can synthesize P(3HB-*co*-3HHx) with desired 3HHx molar composition and have high *M*_w_ in a single large PHA granule. This study successfully engineered PhaC with the desired properties by truncating the N-terminal region of PhaC_BP-M-CPF4_. In a previous study by Lim and co-workers, N-terminal truncation of PhaC from *Aquitalea pelogenes* USM4 (PhaC_*Ap*_) resulted in a significant decrease in the number of PHA granules as well [36, 50]. This observation provided an impetus to this study whereby a more versatile PhaC discovered from mangrove soil metagenome was subjected to further improvement.

Table 3 showed that as more α-helices were truncated in PhaC_BP-M-CPF4_ A27, T74 and D104 mutants, there were greater reduction in PHA content of the bacterial cells. Generally, PhaCs were reported to exist in a monomer–dimer equilibrium, with monomeric form regarded as the inactive form, and dimeric form is regarded as the active form of PhaC [51, 52]. Extensive truncation of the N-terminal region of PhaC may interfere with the dimer formation, resulting in reduced or diminished enzymatic activity of the truncated PhaCs [36]. However, truncation of the first seven amino acids in the N-terminal of PhaC_BP-M-CPF4_ G8 did not affect the amount of PHA accumulated. There was no significant difference in the PHA accumulated by full-length PhaC_BP-M-CPF4_ and G8 although N-terminal truncated PhaCs generally tend to exhibit lower in vitro activities [36, 53]. The in vivo PHA accumulation experiments extended over 48 h, which is a significantly longer duration than the 10–30 min timeframe typically for in vitro enzyme assays. As a result, the reduced activities of PhaC_BP-M-CPF4_ G8 were compensated by the extended accumulation duration in the in vivo experiments using *C. necator* PHB^−^4 transformant.

The 3HHx molar composition of P(3HB-*co*-3HHx) reduced from 6 to 1 mol% as more N-terminal regions were truncated, suggesting that the loss of N-terminal region gradually lost its substrate specificity towards 3HHx. This showed that the N-terminal region of the PhaC played an important role in determining the substrate specificity of the PhaC as reported previously. For instance, originally PhaC_*Cn*_ cannot incorporate any medium-chain-length-PHA (MCL-PHA) monomers into its PHA produced. On the contrary, chimeric PhaCs of PhaC_*Cn*_ with N-terminal region of PhaC_*Ac*_ and PhaC_*Pa*_ were reported able to incorporate MCL-PHA monomers such as 3HHx, 3-hydroxyoctanoate (3HO), and 3-hydroxydecanoate (3HD) [35, 54]. Lim and co-workers also reported similar results as N-terminal truncated PhaC_*Ap*_ too incorporated lesser 3HHx monomers into its PHA [36]. These observations hinted that the N-terminal of PhaCs can affect the PHA monomers incorporated in PHA.

Besides that, there was also an increase in the *M*_w_ of the P(3HB-*co*-3HHx) accumulated by the N-terminal truncated PhaC_BP-M-CPF4_ G8 and A27 compared to the full-length PhaC_BP-M-CPF4_ (Table 3). Previous studies had shown that the 3HHx molar composition in P(3HB-*co*-3HHx) can affects the *M*_w_ of the P(3HB-*co*-3HHx) produced [2, 4]. An increase in 3HHx molar composition will decrease the *M*_w_ of the P(3HB-*co*-3HHx) produced because the 3HHx monomer is a bulky monomer and the increased in the composition of 3HHx will decrease the *M*_w_ which was in agreement with the result obtained in this study [2, 55]. To confirm the finding that N-terminal truncation on PhaCs can increase the *M*_w_ of the PHA produced as observed, the *M*_w_ of P(3HB) homopolymer produced from full-length PhaC_BP-M-CPF4_ and its N-terminal truncated mutants were compared as there was no longer the effect of 3HHx molar composition on the *M*_w_ of the PHA produced. As a result, there was also an increase in the *M*_w_ of P(3HB) produced by the N-terminal truncated PhaC_BP-M-CPF4_ G8 and A27 compared to the full-length PhaC_BP-M-CPF4_ which further supported the finding that N-terminal truncation of PhaC can increase the *M*_w_ of PHA produced. In addition, the *M*_w_ of the PHA produced from fructose was higher compared to that from CPKO. One reason for the lower *M*_w_ of PHA produced by CPKO was probably the presence of glycerol in the culture medium. Glycerol and fatty acids are produced when CPKO is hydrolyzed by lipase. The resulting free fatty acids are used as carbon source by the bacterium for growth and PHA biosynthesis. However, glycerol is poorly used by *C. necator* and had been reported to act as a PHA chain transfer agent which leads to early chain termination of the PHA polymerization, resulting in lower *M*_w_ of PHA produced from CPKO compared to fructose [56].

To determine how N-terminal truncation on PhaCs can increase the *M*_w_ of the PHA produced, gene expression of the full-length PhaC_BP-M-CPF4_ and its N-terminal truncated mutants were verified using qPCR analysis. There were reports on the concentration or the expression of PhaCs affecting the *M*_w_ of the PHA produced [5, 15, 57]. Based on Fig. 2, there was a drop of more than 80% in the gene expression level of the N-terminal truncated PhaC_BP-M-CPF4_ G8 compared to the full-length of PhaC_BP-M-CPF4_ and followed by similarly low expression of PhaC_BP-M-CPF4_ A27, T74, and D104. Lower expression of the N-terminal truncated PhaC will lead to the reduction in the concentration of PhaCs available for polymerizing the PHA monomers into the PHA polymer chains [15, 57]. The decrease in concentration of available PhaCs will increase the ratio of PHA monomers to the concentration of PhaCs in the bacterial cells. The increase in the ratio of PHA monomers to the concentration of PhaCs in the bacterial cells will allow more PHA monomers to be shared among the lower concentration of PhaCs polymerizing the PHA monomers into even longer PHA polymer chains and leading to a higher *M*_w_ of the PHA produced.

In contrast, for PhaC_BP-M-CPF4_ T74, there was a drop in the *M*_w_ of the PHA produced despite having even lower expression. This might be due to the cumulative effect of lower activity and the unstable dimeric structure of PhaC_BP-M-CPF4_ T74. This was also in agreement with the low PHA content accumulated in *C. necator* PHB^−^4 harbouring PhaC_BP-M-CPF4_ T74 in Table 3. The truncated first 73 amino acids might have amino acid residues or segments of amino acids important for the dimerization of the PhaC [36].

**Fig. 4:**
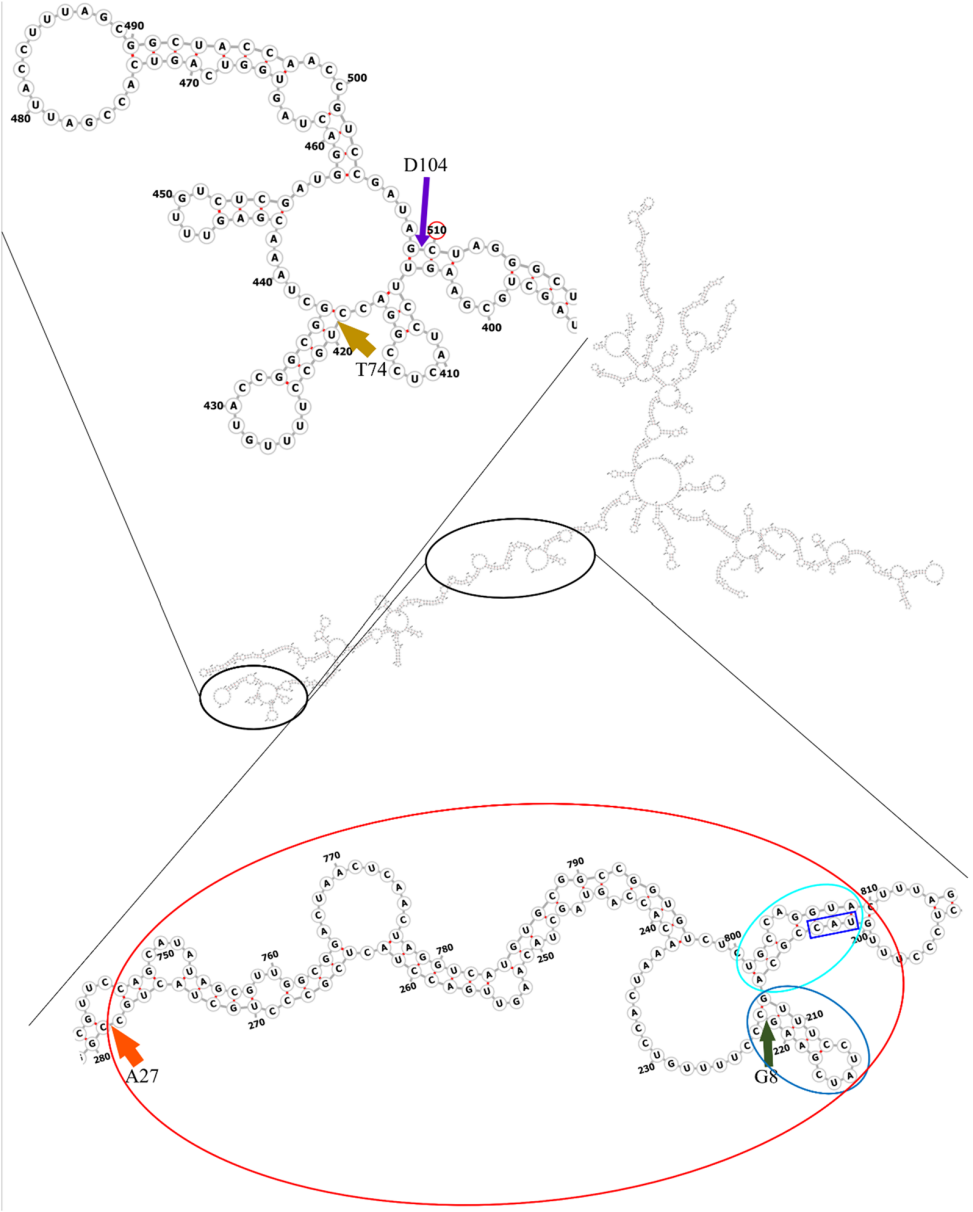
Predicted secondary structure of transcribed mRNA for full-length *phaC*_BP-M-CPF4_. The predicted secondary structure of transcribed mRNA starts from the predicted transcription start site. The start codon was indicated using blue square. The cyan and blue oval indicated stem-loop structures formed by nucleotides which encoded for the first seven amino acids while the red oval indicated the stem-loops structures formed by the nucleotide encoded for the first 26 amino acids residues. Points of truncation for G8, A27, T74, and D104 were indicated using green, orange, brown, and purple arrows respectively

The decrease in gene expression level might be due to the instability of the respective mRNAs in the bacterial cells. Based on the qPCR analysis, the N-terminal regions of the full-length *phaC*_BP-M-CPF4_ seem to provide shielding effect towards mRNA degradation. Figure 4 showed the predicted secondary transcribed mRNA structure of *phaC*_BP-M-CPF4_ starting from the predicted transcription start site. In the predicted secondary mRNA structure, there were numerous stem-loops formed by the nucleotides in the N-terminal regions indicating the participation of the N-terminal region in the formation of stem-loop structures. The stem-loop structures of the mRNA secondary structure was suggested to have the ability to stabilize the mRNA and hence, shielding the mRNA from the mRNA degradation pathways [58]. Based on Fig. 4, the nucleotides that coded for the first seven amino acids of the N-terminal formed were paired with nucleotide 801 to 809 as indicated by cyan oval. Besides that, the nucleotide truncated too formed a stem-loop structure among themselves as indicated with blue oval. Adjacent to the stem-loop structure, there were A/U rich regions which were very susceptible to mRNA degradation by RNase E [59]. The loss of the stem-loop structures probably made the A/U rich region more susceptible to mRNA degradation, leading to a major drop of more than 80% in the gene expression level. For PhaC_BP-M-CPF4_ A27, truncation of first 26 amino acids of the N-terminal removed nucleotides pairing with nucleotide 746 to 809 as indicated with red oval which further compromising the stability of the mRNA leading to even lower gene expression level. For PhaC_BP-M-CPF4_ T74 and D104, truncation points at their respective positions too compromised the ability of the mRNA to form stem-loop structures leading to very low gene expression level. Based on the predicted secondary mRNA structure, it was observed that stem-loop structures indicated in cyan, blue, and red oval played a major role in stability of the mRNA as the loss of both stem-loops heavily affected the mRNA stability as observed PhaC_BP-M-CPF4_ A27, T74 and D104. However, further studies are necessary to further confirm this observation.

One interesting observation was that despite the 5’-untranslated region (UTR) forming many stem-loops structures as observed in Fig. 4, it did not provide sufficient shielding effect from the mRNA degradation pathways. One mRNA degradation pathway which was well-known to play a significant role in mRNA degradation in *E. coli* was the 5’-end-dependent mRNA degradation pathway where the degradation of the mRNA was initiated from the 5’ regions of the mRNA [60]. Previously it was reported that 5’ UTR played major role in determining the stability of mRNA in bacteria cells by shielding the mRNAs from mRNA degradation catalyzed by RNA pyrophosphohydrolase followed by RNase E or RNase J [61–63]. However, this was not observed in our case because the promoter and 5’ UTR of the full-length PhaC_BP-M-CPF4_ and all its N-terminal truncated mutants were the same and there should not be any differences in the gene expression level between the PhaCs. But based on Fig. 2, there were significant drops in the gene expression level for all N-terminal truncated mutants although they had similar promoter, 5’ UTR sequences and the only difference were the missing N-terminal regions. This hinted that the stem-loop structures formed by the missing nucleotide which code for the N-terminal of coding region might play a significant role as well in its respective mRNA stability.

On the bright side, the decrease in expression did not affect the amount of PHA accumulated in the bacterial cells for PhaC_BP-M-CPF4_ G8 as shown in Table 3. Initially, we hypothesized that lower expression of *phaC*s will lead to a lower amount of PHA accumulated in the bacterial cells but that was not the case for PhaC_BP-M-CPF4_ G8. This also showed that for the bacteria to accumulate a high amount of PHA, overexpression of *phaC*s was not necessary as it might lead to a lower *M*_w_ of the PHA produced [5]. To the best of our knowledge, to date, there are no existing reports on the modification of the N-terminal of PhaC or DNA sequence of the PhaC on the expression of the *phaC*s during PHA production.

The increase in *M*_w_ of the PHA produced by N-terminal modified PhaCs was also previously reported by Ye and co-workers where it was reported that deletion of 2 to 65 amino acids in the N-terminal of PhaC from *P. stutzeri* 1317 increased the *M*_w_ of the PHA produced because the α-helices in the N-terminal was predicted to be involved in PHA chain elongation as the PHA chain elongation and termination were easily affected by the changes in the configurational and hydrophilicity of the N-terminal region of PhaCs [33]. The increase in *M*_w_ was also reported to be associated with the changes in the secondary structure of the PhaC and the protein-to-protein interactions. Similar results were also reported by Zheng and co-workers where mutation on the N-terminal affected the interaction between PhaC-to-PhaC and other PHA-related proteins which lead to the increase in *M*_w_ of the PHA accumulated [34]. Kim and co-workers also reported that the deletion of a region at the N-terminal of PhaCs affected the ability of the N-terminal to fold at a proper orientation which could result in the activity of PhaC [53].

Another criterion of a good PhaC for production P(3HB-*co-*3HHx) is the ability to accumulate single large PHA granules as it will make the PHA extraction and purification process easier by using non-solvent PHA extraction methods like centrifugation, filtration, or sedimentation [25, 26]. These non-solvent PHA extraction methods were more straightforward and safer compared to solvent PHA extraction as they were non-toxic and non-flammable.

Based on Fig. 3, there were more single larger PHA granules formed for N-terminal truncated PhaC_BP-M-CPF4_ G8 and A27 compared to full-length PhaC_BP-M-CPF4_. This may be due to the interaction of the full-length PhaC_BP-M-CPF4_ and its N-terminal truncated mutants with other PHA-related proteins like PhaP which is known to be present on the surface of PHA granules and also reported to affect the number and size of PHA granules [9, 26, 64–66]. To date, the most common strategy for the formation of a single granule in the bacterial cell is by deletion of *phaP*s [26, 65, 66]. Previously, a similar observation was obtained through N-and C-terminal truncation of PhaC_*Ap*_ [36, 50]. But the stark difference in the PHA granule morphology was that even the full-length PhaC_BP-M-CPF4_ accumulated lesser PHA granules compared to the full-length PhaC_*Ap*_. To date, there was still no knowledge regarding the origin of this PhaC_BP-M-CPF4_ which was isolated from mangrove soil metagenome, unlike PhaC_*Ap*_ where we know that it comes from *A. pelogenes* USM4 [8, 50].

**Fig. 5:**
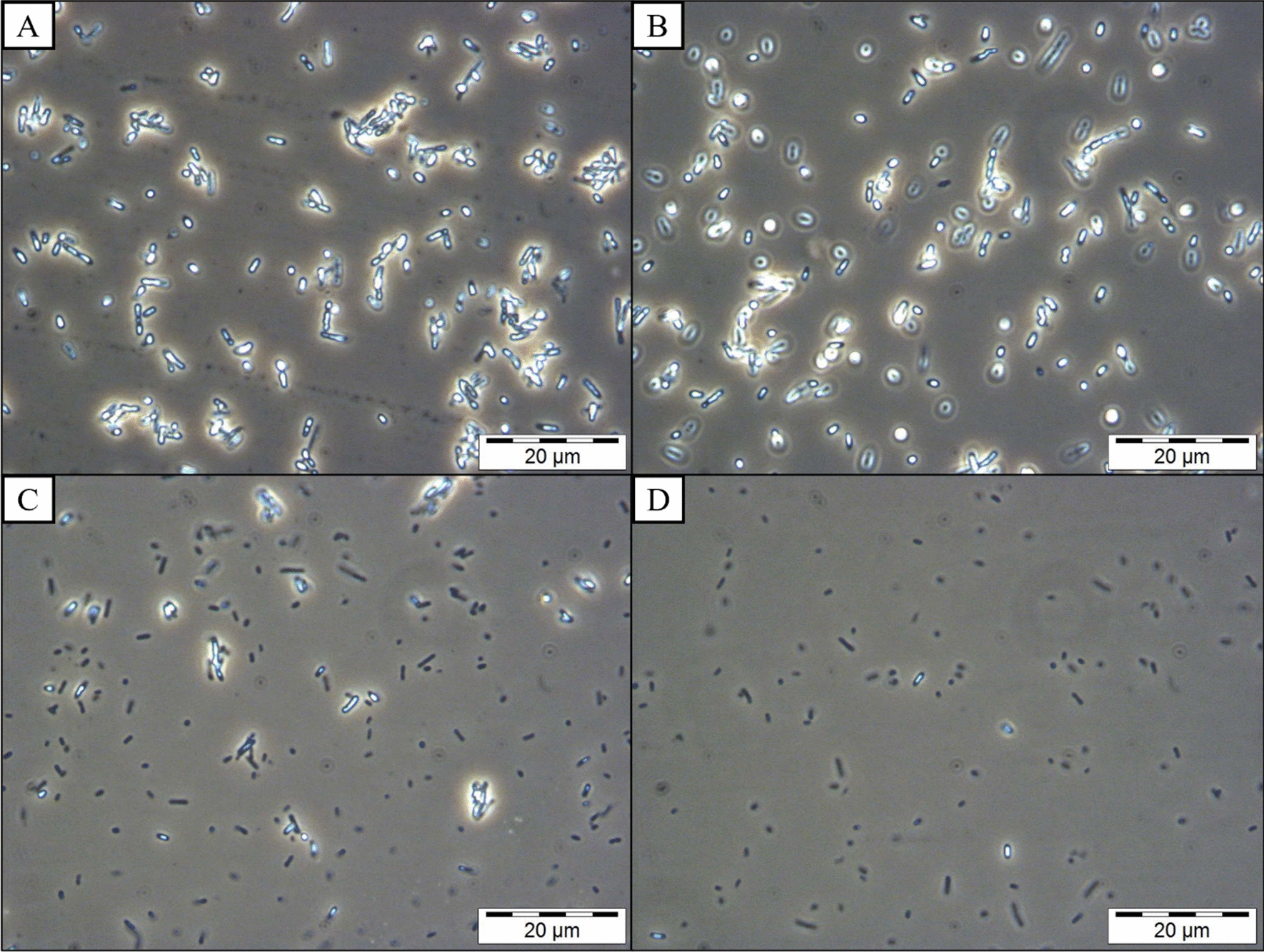
Phase contrast microscopy image of *C. necator* PHB^−^4 harbouring full-length PhaC_BP-M-CPF4_ (**A**), G8 (**B**), A27 (**C**), and T74 (**D**), Magnification: 1000×

Figure 3 also showed that besides accumulating lesser but larger PHA granules, another clear observation was the decrease in the percentage of PHA-containing bacterial cells. As more N-terminal regions of the full-length PhaC_BP-M-CPF4_ were truncated, there were more non-PHA-containing bacteria cells. In Fig. 3A, even with full-length PhaC_BP-M-CPF4_, there were a few bacteria cells in which no PHA granules were observed, but that could be due to the angle of the slide sectioned during ultramicrotome. In Fig. 3C and D, a significant increase in the percentage of bacterial cells without any PHA granules were observed from a cross-section of the bacterial cells. These observations were further confirmed using phase contrast microscopy as shown in Fig. 5. In Fig. 5A and B, the phase contrast images of *C. necator* PHB^−^4 harbouring full-length PhaC_BP-M-CPF4_ and G8 did not show any non-PHA-containing bacteria cells. This showed that the non-PHA-containing cells observed in Fig. 3A and B could be due to the sectioning of the part of the bacteria which did not contain PHA. On the other hand, based on Fig. 5C and D, there was an obvious decrease in the number of PHA-containing bacterial cells from *C. necator* PHB^−^4 harbouring PhaC_BP-M-CPF4_ A27 and T74. These observations were also in agreement with the TEM images observed in Fig. 3C and D where there were increment in the number of non-PHA-containing cells. These observations also agreed with the gene expression level data whereby there were very low expression of the N-terminal truncated mutant PhaC_BP-M-CPF4_ A27 and T74 in the bacteria. As mentioned previously, this might be due to the instability of the mRNA of the N-terminal truncated PhaC_BP-M-CPF4_ A27 and T74. Another possible reason for the absence of PHA granules in the bacterial cells was the difficulty for the N-terminal truncated PhaC_BP-M-CPF4_ A27 and T74 to exist in the dimer form and very low activity of the N-terminal truncated PhaC mutants due to the missing N-terminal region [36]. The lack of PHA granules can also be due to the absence of interaction between the N-terminal truncated PhaC mutants with PhaM which might act as activators for the PhaC polymerization activity [53, 67]. This showed that when bioengineering a potential strain for PHA production, the stability of the mRNAs encoding for PHA-related proteins and their interactions with one another must also be taken into consideration to prevent unnecessary non-PHA-containing bacterial cells.

An interesting phenomenon was observed in Fig. 3B. Some of the PHA material appeared to be flowing/oozing out of the PHA granules of *C. necator* PHB^−^4 harbouring PhaC_BP-M-CPF4_ G8. Such fluidic/mobile nature of PHA in vivo has been reported before. PHA in vivo is maintained in an amorphous and mobile state by bacteria [68]. This amorphous and mobile state is important for the function of PHA as an intracellular carbon-storage compound because the intracellular PHA depolymerase is active only towards non-crystalline PHA. The oozing out of the PHA from its granules may be due to the damage to the surface of the PHA granules during sample preparation for TEM. This observation was only made in *C. necator* PHB^−^4 harbouring PhaC_BP-M-CPF4_ G8 and could be due to the lesser proteins on the granule surface which led to thinner or weaker PHA granule boundaries and hence, easier for the amorphous PHA to ooze out during sample preparation for TEM.

PHA recovery strategy by secretion of intracellular HlyA-signal-peptide-tagged-PHA granule into the culture medium through type I secretion by recombinant *E. coli* from bacterial culture was reported [69]. Based on the observation in Fig. 3B, there is a possibility that the oozing out of PHA from the bacteria cells could be a novel method of PHA recovery from bacterial culture. Mechanical disruption can be applied to rupture the bacterial cell wall and PHA granule boundaries, allowing the PHA to ooze out into the culture medium at the end of cultivation. However, more studies are necessary to confirm this speculation.

Based on all the data discussed above, it is evident that N-terminal truncated PhaC_BP-M-CPF4_ G8 showed improved properties for production of P(3HB-*co*-3HHx). Table 3 showed that the transformant harbouring PhaC_BP-M-CPF4_ G8 produced a higher *M*_w_ of P(3HB-*co*-3HHx) compared to the transformant harbouring the full-length PhaC_BP-M-CPF4_ due to its lower expression without compromising the PHA accumulating ability. The production of P(3HB-*co*-3HHx) with higher *M*_w_ is important as it will contribute to better physical and mechanical properties. The 3HHx molar composition only dropped by 1 mol% which will not have much effect on the physical and mechanical properties of the P(3HB-*co*-3HHx) produced. Another improved property of the N-terminal truncated PhaC_BP-M-CPF4_ G8 was that it accumulated mostly single larger PHA granules compared to the full-length PhaC_BP-M-CPF4_. The production of single larger PHA granules is important as it will facilitate the downstream process of non-solvent PHA extraction and purification.

## Conclusions

In conclusion, the N-terminal of PhaC_BP-M-CPF4_ affected the substrate specificity of the PhaC, molecular weights of the PHA produced, expression of PhaCs, and the granule morphology of the PHA accumulated. N-terminal truncation decreases the 3HHx molar composition of P(3HB-*co*-3HHx), increases the *M*_w_ of the PHA produced, decreases the number and increases the mean diameter of PHA granules accumulated in the bacteria cells. The increase in *M*_w_ was probably due to lower expression of the N-terminal truncated PhaC_BP-M-CPF4_ mutants. Based on the result, among the N-terminal truncated PhaC_BP-M-CPF4_ mutants generated, PhaC_BP-M-CPF4_ G8 showed improved properties of the PHA produced compared to full-length PhaC_BP-M-CPF4_. This study also demonstrated N-terminal truncation can be considered as an alternative approach to improve existing PhaCs in the production of higher *M*_w_ PHA in mostly single and larger PHA granules.

### Supplementary Information

**Additional file 1: Fig. S1**: Predicted N-terminal structure of PhaCBP-M-CPF4. Predicted N-terminal structure of PhaCBP-M-CPF4 using AlphaFold2 program. The colours in the predicted structure represented the level of confidence in the prediction. The black circle indicated the predicted structure has low confidence. The lines indicated where the truncation point of the N-terminal (A). Predicted N-terminal structure of PhaCBP-M-CPF4 using PSIPRED server. The small gap in the α1 helix is indicated with an arrow (B). Predicted secondary structure of the N-terminal of PhaCBP-M-CPF4 using a combination of AlphaFold2 program and PSIPRED server. The bold alphabets indicate the structure predicted using the PSIPRED server while the unbold alphabets indicate the structure predicted using Alphafold2 program. The truncation points were indicated with black lines (C). **Fig. S2**: Primer efficiency for 16S rRNA (A), *phaC*BP-M-CPF4 (B) primers. **Fig. S3**: Melt curve analysis for 16S rRNA (A), *phaC*BP-M-CPF4 (B) primers.

## Data Availability

All the data in this study are included in this published article and its additional files.

## Abbreviations

PHA: Polyhydroxyalkanoate
P(3HB): Poly(3-hydroxybutyrate)
P(3HB*-co-*3HHx): Poly[(*R*)*-*3-hydroxybutyrate*-co-*(*R*)*-*3-hydroxyhexanoate]
3HB: 3-Hydroxybutyrate
MCL-PHA: Medium-chain-length-PHA
3HHx: 3-Hydroxyhexanoate
3HO: 3-Hydroxyoctanoate
3HD: 3-Hydroxydecanoate
PhaC: PHA synthase
*M*_n_: Number-average molecular weight
*M*_w_: Weight-average molecular weight
PDI: Polydispersity index
LDPE: Low-density polyethylene
PP: Polypropylene
RBS: Ribosome binding site
PhaC_BP-M-CPF4_: PhaC isolated from mangrove soil metagenome
PhaC_*C*__s_: PhaC from *Chromobacterium* sp. USM2
PhaC_*Ra*_: PhaC from *Rhodococcus aetherivorans* I24
PhaC_*Ah*_: PhaC from *Aeromonas hydrophila* 4AK4
PhaC_*Ac*_: PhaC from *Aeromonas caviae*
PhaC_*Cn*_: PhaCs from *Cupriavidus necator* H16
PhaC_*Pa*_: PhaC from *Pseudomonas aeruginosa*
PSI-BLAST: Position-Specific Iterative Basic Local Alignment Search Tool
PSIPRED: PSI-blast based secondary structure prediction
NR: Nutrient-rich
MM: Mineral medium
CPKO: Crude palm kernel oil
PCR: Polymerase chain reaction
NCBI: National Center for Biotechnology Information
GC: Gas chromatography
CME: Caprylic acid methyl ester
GPC: Gas permeation chromatography
HPLC: High-performance liquid chromatography
qPCR: Quantitative polymerase chain reaction
RNA: Ribonucleic acid
mRNA: Messenger RNA
UTR: Untranslated region
EDTA: Ethylenediaminetetraacetic acid
cDNA: Complementary deoxyribonucleic acid
Ct: Cycle threshold
TEM: Transmission electron microscopy
OsO_4_: Osmium tetroxide
ANOVA: Analysis of variance
pLDDT: Per-residue estimate of its confidence
CDW: Cell dry weight
