# Supplementary material for: N-terminal truncation of PhaCBP-M-CPF4 and its effect on PHA production
Source: Microb Cell Fact. 2024 Feb 15;23:52. doi: 10.1186/s12934-024-02329-w (PMC10867992; doi:10.1186/s12934-024-02329-w)
Supplement: Supplementary file 1 — Additional file 1: Fig. S1: Predicted N-terminal structure of PhaCBP-M-CPF4. Predicted N-terminal structure of PhaCBP-M-CPF4 using AlphaFold2 program. The colours in the predicted structure represented the level of confidence in the prediction. The black circle indicated the predicted structure has low confidence. The lines indicated where the truncation point of the N-terminal (A). Predicted N-terminal structure of PhaCBP-M-CPF4 using PSIPRED server. The small gap in the α1 helix is indicated with an arrow (B). Predicted secondary structure of the N-terminal of PhaCBP-M-CPF4 using a combination of AlphaFold2 program and PSIPRED server. The bold alphabets indicate the structure predicted using the PSIPRED server while the unbold alphabets indicate the structure predicted using Alphafold2 program. The truncation points were indicated with black lines (C). Fig. S2: Primer efficiency for 16S rRNA (A), phaCBP-M-CPF4 (B) primers. Fig. S3: Melt curve analysis for 16S rRNA (A), phaCBP-M-CPF4 (B) primers. [file 12934_2024_2329_MOESM1_ESM.pdf]

**Additional File**

**N-terminal truncation of $PhaC_{BP\text{-}M\text{-}CPF4}$ and its effect on PHA production**

Submitted for publication to Microbial Cell Factories

Soon Zher Neoh [a], Hua Tiang Tan [b], Chanaporn Trakunjae [c], Min Fey Chek [b], Pilanee Vaithanomsat [c], Toshio Hakoshima [b], and Kumar Sudesh [a,*]

[a] Ecobiomaterial Research Laboratory, School of Biological Sciences, Universiti Sains Malaysia, 11800 USM, Pulau Pinang, Malaysia

[b] Structural Biology Laboratory, Nara Institute of Science and Technology, 8916-5 Takayama, Ikoma, Nara 630-0192, Japan

[c] Kasetsart Agricultural and Agro-Industrial Product Improvement Institute (KAPI), Kasetsart University, Bangkok 10900, Thailand

*Corresponding author:

Kumar Sudesh

School of Biological Sciences

Universiti Sains Malaysia

11800 Penang, Malaysia

Tel: +604 6534367

Fax: +604 6565125

Email: ksudesh@usm.my

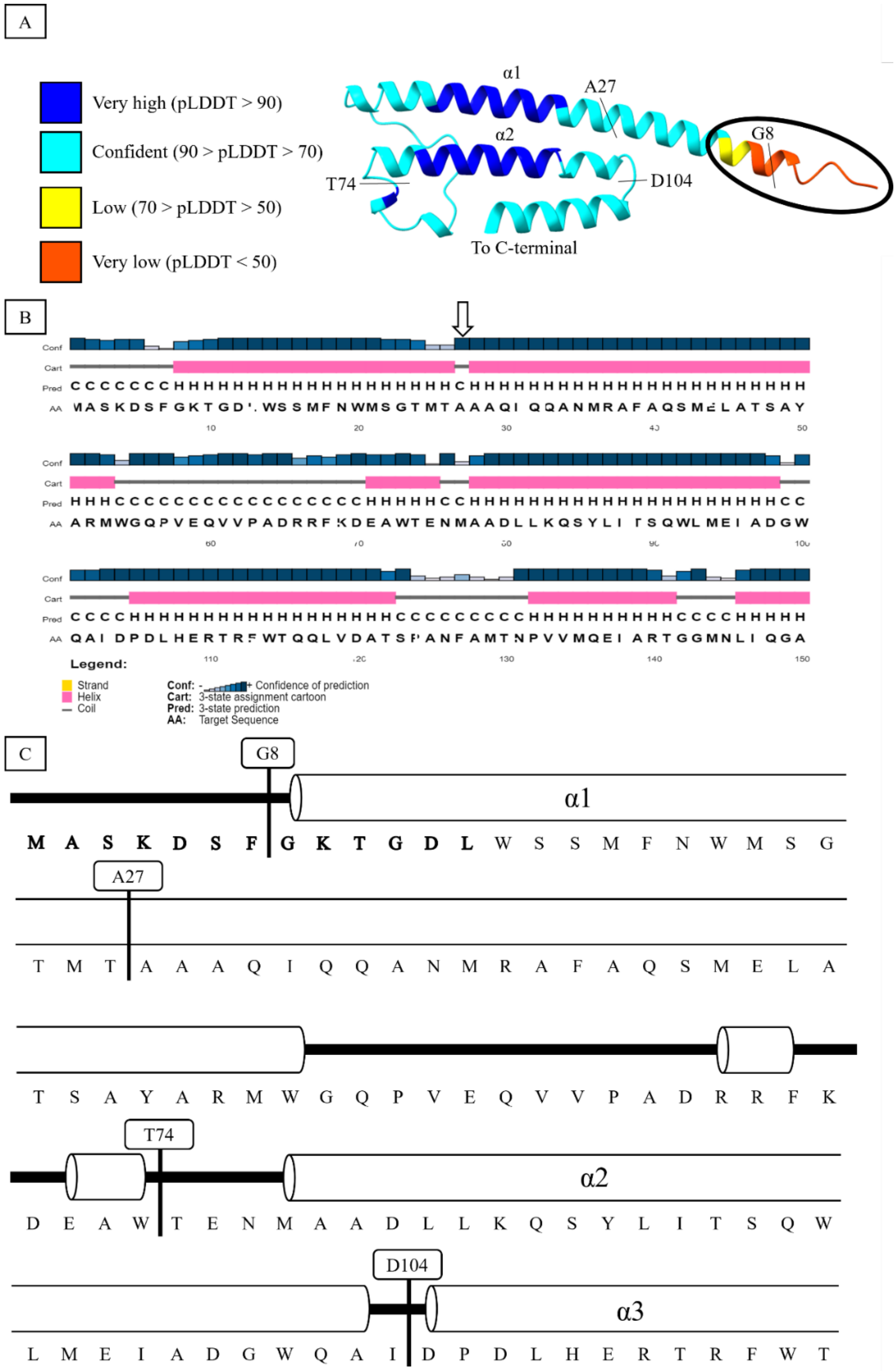


**Fig. S1**: Predicted N-terminal structure of PhaC$_{BP\text{-}M\text{-}CPF4}$. Predicted N-terminal structure of PhaC$_{BP\text{-}M\text{-}CPF4}$ using AlphaFold2 program. The colours in the predicted structure represented the level of confidence in the prediction. The black circle indicated the predicted structure has low confidence. The lines indicated where the truncation point of the N-terminal (A). Predicted N-terminal structure of PhaC$_{BP\text{-}M\text{-}CPF4}$ using PSIPRED server. The small gap in the α1 helix is indicated with an arrow (B). Predicted secondary structure of the N-terminal of PhaC$_{BP\text{-}M\text{-}CPF4}$ using a combination of AlphaFold2 program and PSIPRED server. The bold alphabets indicate the structure predicted using the PSIPRED server while the unbold alphabets indicate the structure predicted using Alphafold2 program. The truncation points were indicated with black lines (C).

A

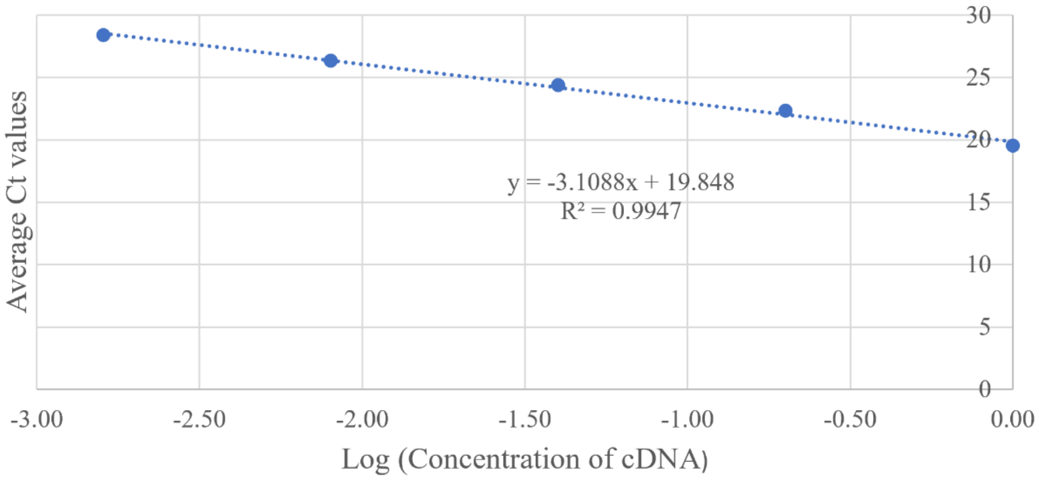


$$\text{Primer efficiency} = (10^{(-1/\text{slope})} - 1) \times 100\%$$
$$= (10^{(-1/-3.1088)} - 1) \times 100\%$$
$$= 109\%$$

B

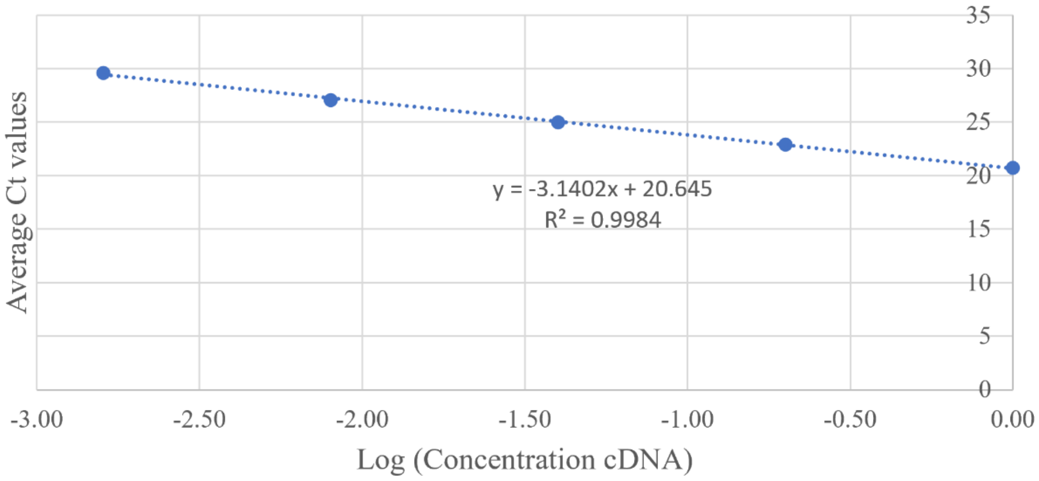


$$\text{Primer efficiency} = (10^{(-1/\text{slope})} - 1) \times 100\%$$
$$= (10^{(-1/-3.1402)} - 1) \times 100\%$$
$$= 108\%$$

**Fig. S2**: Primer efficiency for 16S rRNA (A), *phaC*$_{\text{BP-M-CPF4}}$ (B) primers.

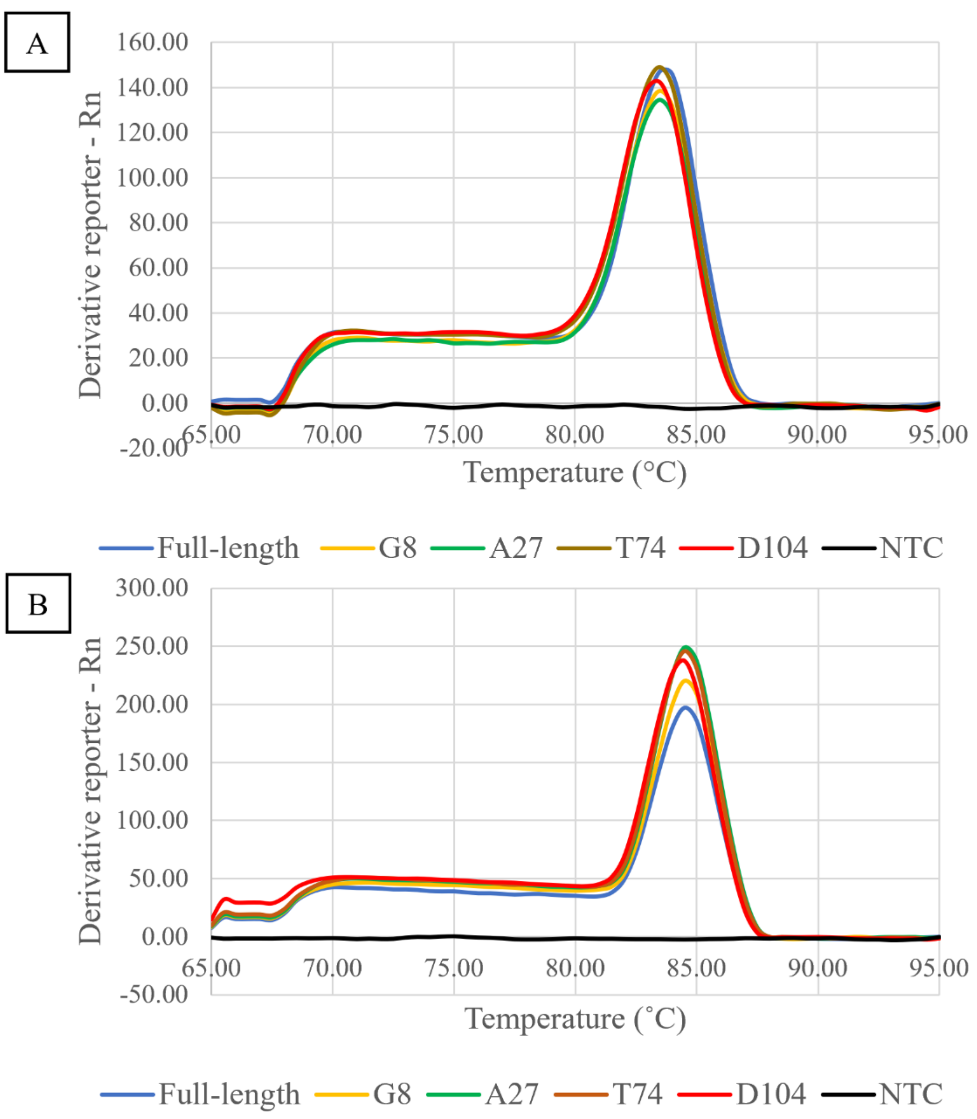


**Fig. S3**: Melt curve analysis for 16S rRNA (A), *phaC*$_{BP\text{-}M\text{-}CPF4}$ (B) primers.
